# Association Between the Incidence of Pancreatic Fistula After Pancreaticoduodenectomy and the Degree of Pancreatic Fibrosis

**DOI:** 10.1007/s11605-017-3660-2

**Published:** 2018-01-12

**Authors:** Yong Deng, Baixiong Zhao, Meiwen Yang, Chuanhong Li, Leida Zhang

**Affiliations:** Department of Hepatobiliary Surgery, Southwest Hospital, Third Military Medical University (Army Medical University), No. 30, GaoTanYan Street, Chongqing, 400038 People’s Republic of China

**Keywords:** Pancreaticoduodenectomy, Pancreatic fistula, Fibrosis

## Abstract

**Objective:**

The objective of this study is to investigate the association between the incidence of pancreatic fistula after pancreaticoduodenectomy (PD) and the degree of pancreatic fibrosis.

**Method:**

Between January 2013 and December 2016, the analysis of the clinical data of 529 cases of pancreaticoduodenectomy patients of our hospital was performed in a retrospective fashion. The univariate analysis and multivariate analysis were done using the Pearson chi-squared test and binary logistic regression analysis model; correlations were analyzed by Spearman rank correlation analysis. The value of the degree of pancreatic fibrosis to predict the incidence of pancreatic fistula after pancreaticoduodenectomy was evaluated by the area under the receiver operating characteristic (ROC) curve.

**Results:**

The total incidence of pancreatic fistula after pancreaticoduodenectomy was 28.5% (151/529). Univariate analysis and multivariate analysis showed that BMI ≥ 25 kg/m^2^, pancreatic duct size ≤ 3 mm, pancreatic CT value< 30, the soft texture of the pancreas (judged during the operation), and the percent of fibrosis of pancreatic lobule ≤ 25% are prognostic factors of pancreatic fistula after pancreaticoduodenectomy (*P* < 0.05); the pancreatic CT value and the percent of fibrosis of pancreatic lobule in pancreatic fistula group were both lower than those in non-pancreatic fistula group (*P* < 0.05). Results indicated that there is a negative correlation between the severity of pancreatic fistula and the pancreatic CT value or the percent of fibrosis of pancreatic lobule (*r* = − 0.297, − 0.342, respectively). The areas under the ROC curve of the percent of fibrosis of pancreatic lobule and the pancreatic CT value were 0.756 and 0.728, respectively.

**Conclusion:**

The degree of pancreatic fibrosis is a prognostic factor which can influence the pancreatic texture and the incidence of pancreatic fistula after pancreaticoduodenectomy. The pancreatic CT value can be used as a quantitative index of the degree of pancreatic fibrosis to predict the incidence of pancreatic fistula after pancreaticoduodenectomy.

## Introduction

Pancreaticoduodenectomy (PD) is one of the most complicated abdominal surgery.^1^ In the past decades, the safety of PD procedure has been greatly improved; however, the incidence of postoperative complications has remained constant during these years. Pancreatic fistula after PD remains one of the intractable complications of PD. It has been estimated that around 50% of postoperative complications after PD are related to pancreatic fistula.^2^^–^4 Pancreatic fistula after PD which is associated with higher mortality and longer postoperative hospital stay can result in abdominal abscess, hemorrhage, and sepsis, and its incidence rate is about 4–5%.^5^ The soft texture of the pancreas is an accepted independent risk factor which can influence the possibility and severity of pancreatic fistula after PD. However, most researchers determine whether the texture of the pancreas is soft or not depending on their subjective experience, lacking in uniform standards.^6^ Fibrosis refers to the pathological progress in which fibrous connective tissue increases and parenchymal cells decrease in organs, usually accompanied by chronic inflammation of the organs and tissues. Pancreatic fibrosis which is a common pathological change of pancreatic tissue often leads to hardening the texture of the pancreas, lacks internationally accepted grading standards.^7^^–^9 It has been reported that preoperative pancreas CT value can predict the incidence of pancreatic fistula after PD.^10^,^11^ Therefore, we assume that the degree of pancreatic fibrosis is a key factor which can influence the incidence of pancreatic fistula after PD, and preoperative pancreas CT value can indirectly reflect the degree of pancreatic fibrosis. We analyzed retrospectively 529 patients treated by the Southwest Hospital between 2013 and 2016 to identify some factors which can influence the incidence of pancreatic fistula after PD and to investigate the correlation between the incidence of pancreatic fistula after PD and the degree of pancreatic fibrosis.

## Materials and Methods

### Materials

Five hundred twenty-nine patients were admitted by the Southwest Hospital between January 2013 and December 2016. Their age ranged from 14 to 82 and the average age is 55.1. Among the above patients, 355 patients were male and 174 were female. Pathological diagnosis showed that there were 348 cases of pancreatic ductal adenocarcinoma (PDAC), 102 cases of cancer originated from gastrointestinal tract, 28 cases of neuroendocrine neoplasm, 25 cases of inflammatory change, 16 cases of pancreatic papilloma, eight cases of serous cystadenoma, and two cases of retroperitoneal liposarcoma (RPLS). All the operations utilized Child procedure to rebuild the digestive tract and were performed by doctors who at least have the title of associate professor.

### Diagnostic Criteria and Grading of Pancreatic Fistula

The diagnostic and including criteria of pancreatic fistula in this research adopted the definition of pancreatic fistula which is given by the International Study Group on Pancreatic Fistula (ISGPF)^12^: drainage fluid of more than 10 mL in 24 h with the amylase at least three times the normal serum activity 3 or 4 days postoperatively. Grading grade A has no clinical impact and requires slight change in management or deviation from the normal clinical pathway; grade B, requires a change in management or adjustment in the clinical pathway, and the patient is often kept with nothing by mouth and is supported with partial or total parental or enteral nutrition; grade C, a major change in clinical management or deviation from the normal clinical pathway occurs. Clinical intervention is aggressive with the patient kept nothing by mouth and total parenteral nutrition or enteral nutrition, intravenous antibiotics, and somatostatin analogues instituted, often in an intensive care unit setting. The patient typically requires an extended hospital stay with a major delay in hospital discharge. This research classified patients after PD therapy as pancreatic fistula group and non-pancreatic fistula group according to this standard, and recorded the grades of pancreatic fistula.

### CT Value and Measurement

CT value is the attenuation value of X-ray after the absorption of the tissue it passed.^13^ CT value is usually used to measure the density of an organ or local tissue in the body. Before the operation, abdominal helical enhancement scanning was performed in patients by a 64-slice dual-source CT (Siemens, Germany), and the thickness is 5 mm. The pancreatic CT value was measured by a medical imaging doctor who did not know our research or patient information. Measurement: on the CT image, a region to be measured was obtained on the left side of where the superior mesenteric artery crosses over of the pancreas; then, the CT value of this area was identified automatically by computer. An average value was calculated based on the data of three levels before and after repeated measurement, which was taken as the final CT value.

### Making and Observing Pathological Section

All the pathological sections in this research were sampled from the remaining pancreatic tissue closing to the incision, and embedded in paraffin. The thickness of all the sections was about 5 μm. Stained by hematoxylin-eosin. Then, the sections were observed by electron microscope.

### Statistical Analysis

Data were statistically analyzed by SPSS 18.0. Univariate analysis was done using the Pearson chi-squared test. Then, the factors which are statistically significant were analyzed by binary logistic regression analysis. The correlation between pancreatic CT value and the percent of fibrosis of pancreatic lobule was analyzed by Spearman rank correlation analysis. We used the area under the ROC curve to evaluate the pancreatic CT value and the percentage of fibrosis of pancreatic lobule to predict the incidence of pancreatic fistula after PD. The area under the ROC curve higher than 0.9 indicates a high predictive value; the area under the ROC curve ranges from 0.7 to 0.9 indicates a moderate predictive value; the area under the ROC curve ranges from 0.5 to 0.7 indicates a low predictive value. Differences were considered as significant at the level of *P* = 0.05.

## Results

### The Incidence of Pancreatic Fistula After PD

Within 1 month after PD, there were 151 cases of pancreatic fistula in 529 patients (28.5%), including 79 cases of grade A pancreatic fistula (14.9%), 60 cases of grade B (11.3%), and 12 cases of grade C pancreatic fistula (2.3%).

### Results of Univariate Analysis

Results of Pearson chi-squared test indicated that the incidence of pancreatic fistula after PD has a correlation with BMI, preoperative γ-GGT, pancreatic duct size, CT value, surgical time, pancreatic texture (judged during the operation), and the percent of fibrosis of pancreatic lobule (*P* < 0.05); however, there is no correlation between the incidence of pancreatic fistula after PD and gender, age, diabetes history, hypertension history, history of abdominal surgery, hemoglobin, albumin, total bilirubin, ALT, AST, alkaline phosphatase, surgical method, intraoperative blood loss, or situation of vessels invaded by carcinoma (*P* > 0.05) (Table 1).

**Table 1:** Results of univariate analysis of 408 cases of pancreatic fistula after PD

Item		Number of cases	Percent of pancreatic fistula (%)	χ^2^ value	*P* value
Gender	Male	355	103 (29.0)	0.117	0.733
	Female	174	48 (27.6)		
Age (year)	< 55	248	69 (27.8)	0.119	0.730
	≥ 55	281	82 (29.2)		
BMI (kg/m^2^)	< 25	404	101 (25.0)	10.531	0.001
	≥ 25	125	50 (40.0)		
Diabetes history	No	468	135 (28.8)	0.181	0.670
	Yes	61	16 (26.2)		
Hypertension history	No	448	126 (28.1)	0.252	0.615
	Yes	81	25 (30.8)		
History of abdominal surgery	No	420	122 (29.0)	0.253	0.615
	Yes	109	29 (26.6)		
Preoperative hemoglobin (g/L)	> 120	271	84 (30.9)	1.638	0.201
	≤ 120	258	67 (26.0)		
Preoperative albumin (g/L)	< 30	19	8 (42.1)	1.777	0.183
	≥ 30	510	143 (28.0)		
Preoperative total bilirubin (μmol/L)	> 171	196	63 (32.1)	1.977	0.160
	≤ 171	333	88 (26.4)		
Preoperative ALT(U/L)	> 126	211	67 (31.8)	1.772	0.183
	≤ 126	318	84 (26.4)		
Preoperative AST(U/L)	> 126	174	52 (29.9)	0.228	0.633
	≤ 126	355	99 (27.9)		
Preoperative γ-GGT(U/L)	> 150	345	110 (31.9)	5.424	0.020
	≤ 150	184	41 (22.3)		
Preoperative alkaline phosphatase (U/L)	> 342	248	77 (31.0)	1.435	0.231
	≤ 342	281	74 (26.3)		
Pancreatic duct size (mm)	> 3	271	38 (14.0)	57.054	0.001
	≤ 3	258	113 (43.8)		
CT value	< 40	96	38 (39.6)	7.007	0.008
	≥ 40	433	113 (26.1)		
Surgical method	PPPD	161	48 (29.8)	0.183	0.669
	PD	368	103 (28.0)		
Surgical time (min)	< 360	215	51 (23.7)	4.132	0.042
	≥ 360	314	100 (31.8)		
Intraoperative blood loss (mL)	< 400	226	70 (31.0)	1.141	0.285
	≥ 400	303	81 (26.7)		
Pancreatic texture (judged during the operation)	Soft	268	114 (42.5)	52.144	0.001
	Hard	261	37 (14.2)		
Situation of vessels invaded by carcinoma	No	459	130 (28.3)	0.084	0.772
	Yes	70	21 (30.0)		
Percent of fibrosis of pancreatic lobule	> 25	283	56 (19.8)	22.877	0.001
	≤ 25	246	95 (38.6)		

### Results of Multivariate Logistic Regression Analysis

Results of multivariate logistic regression analysis suggested that BMI ≥ 25 kg/m^2^, pancreatic duct size ≤ 3 mm, CT value < 40, hard texture of pancreas (judged during the operation), and percent of fibrosis of pancreatic lobule ≤ 25% are prognostic factors of pancreatic fistula after PD (Table 2) .

**Table 2:** Results of multivariate logistic regression analysis of 408 cases of pancreatic fistula after PD

Item	B	S.E.	Wald	*P* value	EXP(B) (95% CI)
BMI (≥ 25 kg/m^2^ or < 25 kg/m^2^)	0.187	0.186	2.033	0.032	1.484 (1.806~2.743)
Preoperative γ-GGT(> 150 U/L or ≤ 150 U/L)	0.142	0.256	0.311	0.231	0.469 (0.644~2.312)
Pancreatic duct size(> 3 mm or ≤ 3 mm)	− 0.368	0.168	4.772	0.021	1.595 (1.545~1.938)
CT value(< 40 or ≥ 40)	− 0.886	0.246	9.821	0.029	1.615 (1.255~1.779)
Surgical time (< 360 min or ≥ 360 min)	0.049	0.212	0.042	0.324	1.011 (0.579~1.562)
Pancreatic texture (judged during the operation) (soft or hard)	− 0.483	0.263	4.978	0.041	2.595 (2.169~2.994)
Percent of fibrosis of pancreatic lobule (> 25% or ≤ 25%)	− 0.911	0.256	12.475	0.033	2.413 (2.265~2.837)

### Comparisons of the CT Values and Percent of Fibrosis of Pancreatic Lobule of Different Grades of Pancreatic Fistula

The differences of the CT values and the percent of fibrosis of pancreatic lobule of different grades of pancreatic fistula were statistically significant (*P* = 0.016, 0.021, respectively). The pancreatic CT values and percent of fibrosis of pancreatic lobule of mild pancreatic fistula patients are higher than those of heavy pancreatic fistula patients, and pancreatic CT value and percent of fibrosis of pancreatic lobule all had a rank correlation with the severity of pancreatic fistula (*P* < 0.01) (Table 3).

**Table 3:** Comparisons of the CT values and percent of fibrosis of pancreatic lobule of different grades of pancreatic fistula ($\overline{x}\pm s$)

Grade of pancreatic fistula	Number of cases	Pancreatic CT value	Percent of fibrosis of pancreatic lobule
A	79	38.8 ± 2.3	26.0 ± 1.3
B	60	34.7 ± 1.9^a^	21.1 ± 1.1^a^
C	12	30.1 ± 2.5^ab^	17.9 ± 1.8^ab^
*F* value	–	8.343	7.601
*P* value	–	0.016	0.021

^a^*P* < 0.05 vs grade A

^b^*P* < 0.05 vs grade B

### The Correlations Between the Severity of Pancreatic Fistula and Pancreatic CT Value or Percent of Fibrosis of Pancreatic Lobule

We assigned non-pancreatic fistula, grade A, grade B, and grade C to 0, 1, 2, 3, respectively. Analyzed by linear correlation analysis, we found that there is a negative correlation between the severity of pancreatic fistula and pancreatic CT value or percent of fibrosis of pancreatic lobule (*r* = − 0.297, *P* < 0.05; *r* = − 0.342, *P* < 0.05).

Spearman rank correlation analysis suggested that there is a high correlation between pancreatic CT value and percent of fibrosis of pancreatic lobule (rank correlation = 0.675, *P* = 0.026).

### The Area Under the ROC Curve

**Fig. 1:**
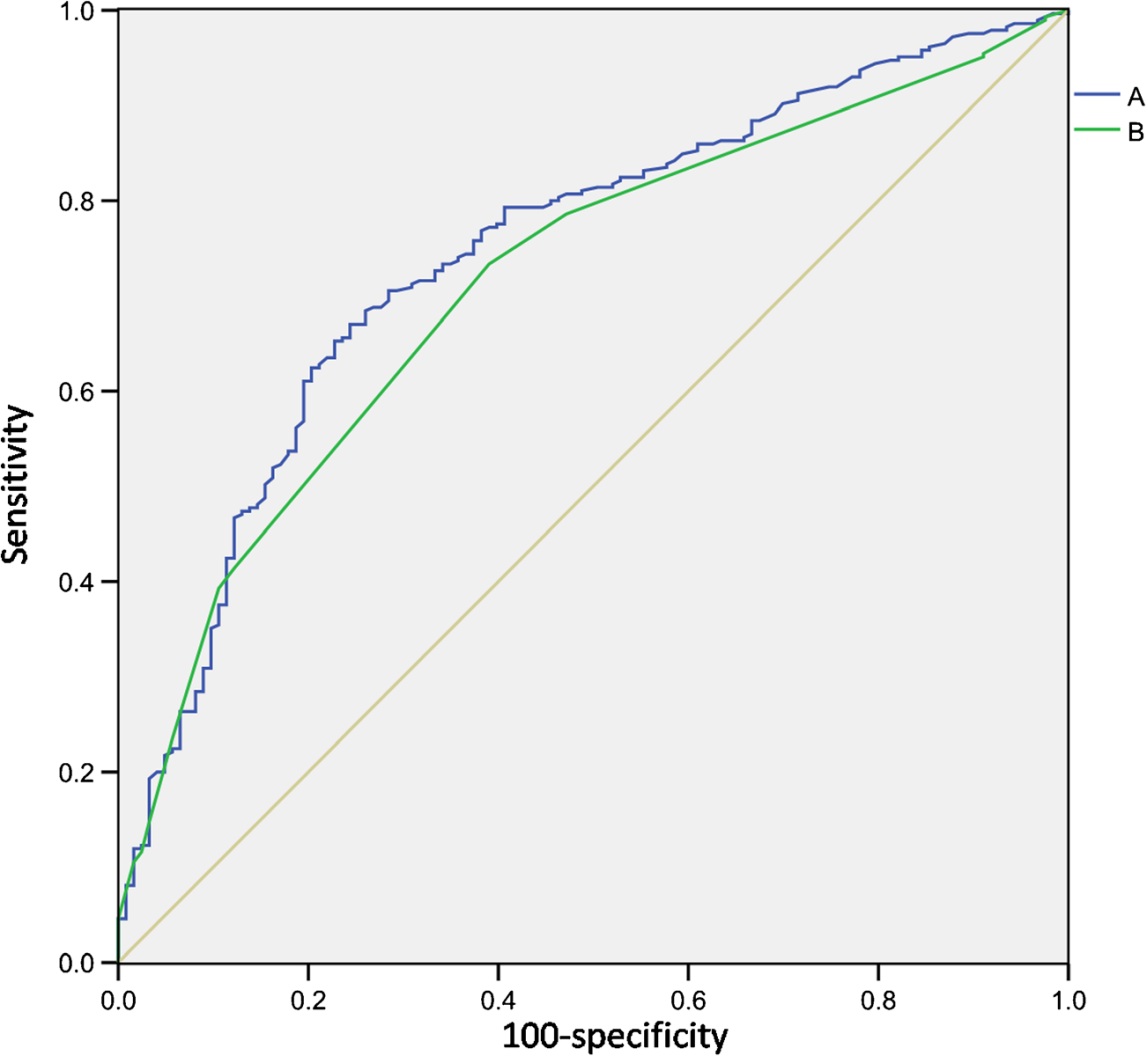
ROC curve of preoperative pancreatic CT value and percent of fibrosis of pancreatic lobule. **a** Percent of fibrosis of pancreatic lobule; **b** Pancreatic CT value

Results showed that the areas under ROC curve are 0.756 and 0.728, respectively. The areas under ROC curve also indicated that preoperative pancreatic CT value and the postoperative percent of fibrosis of pancreatic lobule could be used to predict the incidence of pancreatic fistula after PD and both have a moderate predictive value (95% *CI* ranges from 0.699 to 0.797 and 0.668 to 0.769, respectively; *P* < 0.01) (Fig. 1).

## Discussion

Pancreatic fistula is one of the main causes which lead to other complications and death.^14^,^15^ Recent research suggests that the main cause of pancreatic fistula formation is that normal pancreatic tissues are injured during the PD.^3^ Shubert et al^16^ suggests that preoperative risk evaluation of pancreatic cancer patients can effectively predicate the incidence of pancreatic fistula after PD. Our research found that pancreatic CT value and percent of fibrosis of pancreatic lobule in pancreatic fistula after PD are much lower than those in non-pancreatic fistula after PD, and that pancreatic CT value as well as the percent of fibrosis of pancreatic lobule vary in different grades of pancreatic fistula after PD. The severity of pancreatic fistula is negatively correlated with pancreatic CT value or the percent of fibrosis of pancreatic lobule, and there is a correlation between pancreatic CT value and the percent of fibrosis of pancreatic lobule. The area under ROC curve also suggests that as two quantitative indexes of the degree of pancreatic fibrosis, the percent of fibrosis of pancreatic lobule and the pancreatic CT value both have a moderate role in predicting the incidence of pancreatic fistula after PD. Combined with the above two indexes, the predictive accuracy of the incidence of pancreatic fistula after PD will be largely improved. And both these two indexes have a great significance in instructing the delineation of perioperative therapeutic schemes.

In 1984, Longmire^17^ first proposed that the soft texture of pancreas is one of the prognostic factors of pancreatic fistula after PD. Results of univariable and multivariate logistic regression analysis from this research indicated that the texture of pancreas (judged during the operation) is an independent prognostic factor of pancreatic fistula after PD. Among 268 cases of PD whose pancreatic texture were soft, 114 cases developed pancreatic fistula; however, among 261 cases of PD whose pancreatic texture were hard, only 37 cases developed pancreatic fistula. Postoperative pathological findings showed that the percent of fibrosis of pancreatic lobule can objectively reflect the degree of pancreatic fibrosis from a pathological angle, and it has been reported that if the degree of pancreatic fibrosis is lighter, the percent of fibrosis of pancreatic lobule is often lower than 25%.^18^

At present, most researchers determine whether the texture of the pancreas is soft or not depending on their subjective experience, lacking in objective uniform standards. Although some studies utilized the CT value to evaluate the degree of pancreatic fibrosis, the included cases were too scarce to form a scientific evaluation system.^10^,^11^ CT value refers to the attenuation that rays were absorbed by body after crossing the tissue, and it often represents tissue density clinically.^19^ Thus, the higher the tissue density is, the larger the CT value is. And the normal pancreatic CT value is between 40 and 54. According to this feature, CT values are often used to measure the density of tissues in vivo clinically. Although density is not completely equal to the degree of fibrosis, results of univariable and multivariate logistic regression analysis from this research both strongly proved that CT values of preoperative pancreatic tissues are lower than 40 is an independent prognostic factor of PD. Moreover, different grades of pancreatic fistula have a correlation with the degree of fibrosis, and the severity of pancreatic fistula is negatively correlated with pancreatic CT value and the percent of fibrosis of pancreatic lobule.

After analyzing the data of preoperative pancreatic CT value and postoperative percent of fibrosis of pancreatic lobule, linear regression equation was established, and correlations were examined as well. Results indicated that there is a linear correlation between the above two indexes, so these two indexes both can reflect the degree of pancreatic fibrosis and predict the incidence of pancreatic fistula after PD. In the meantime, results of ROC curve also suggested that preoperative pancreatic CT value and postoperative percent of fibrosis of pancreatic lobule can be used to predict the incidence of pancreatic fistula after PD. Based on the above results, we assumed that preoperative pancreatic CT value and postoperative percent of fibrosis of pancreatic lobule both can be used as objective indexes to reflect the degree of pancreatic fibrosis. Hence, they can be used to predict the incidence of pancreatic fistula after PD. From a practical standpoint, however, results of postoperative pathological examinations cannot instruct the formulation of preoperative therapeutic plan. On the contrary, preoperative pancreatic CT value has a great significance in instructing the formulation of perioperative therapeutic plan, as CT value can be obtained noninvasively and before operation.

In conclusion, our research suggests that BMI, pancreatic duct size, pancreatic CT value, texture of the pancreas, and postoperative percent of fibrosis of pancreatic lobule are prognostic factors of pancreatic fistula after PD. There is a good linear correlation between pancreatic CT value and postoperative percent of fibrosis of pancreatic lobule, so they can be used as objective indexes to reflect the degree of pancreatic fibrosis. It can be more scientifically to predict the incidence of pancreatic fistula after PD if the above two indexes combine with subjective judgment. However, it still needs further research to understand whether there are differences in pancreatic CT value and postoperative percent of fibrosis of pancreatic lobule among cohorts and to determine how to judge the degree of pancreatic fibrosis preoperatively.
